# Deep Neural Networks with Multistate Activation Functions

**DOI:** 10.1155/2015/721367

**Published:** 2015-09-10

**Authors:** Chenghao Cai, Yanyan Xu, Dengfeng Ke, Kaile Su

**Affiliations:** ^1^School of Technology, Beijing Forestry University, No. 35 Qinghuadong Road, Haidian District, Beijing 100083, China; ^2^School of Information Science and Technology, Beijing Forestry University, No. 35 Qinghuadong Road, Haidian District, Beijing 100083, China; ^3^Institute of Automation, Chinese Academy of Sciences, No. 95 Zhongguancundong Road, Haidian District, Beijing 100190, China; ^4^College of Mathematics Physics and Information Engineering, Zhejiang Normal University, No. 688 Yingbin Road, Jinhua 321004, China

## Abstract

We propose multistate activation functions (MSAFs) for deep neural networks (DNNs). These MSAFs are new kinds of activation functions which are capable of representing more than two states, including the *N*-order MSAFs and the symmetrical MSAF. DNNs with these MSAFs can be trained via conventional Stochastic Gradient Descent (SGD) as well as mean-normalised SGD. We also discuss how these MSAFs perform when used to resolve classification problems. Experimental results on the TIMIT corpus reveal that, on speech recognition tasks, DNNs with MSAFs perform better than the conventional DNNs, getting a relative improvement of 5.60% on phoneme error rates. Further experiments also reveal that mean-normalised SGD facilitates the training processes of DNNs with MSAFs, especially when being with large training sets. The models can also be directly trained without pretraining when the training set is sufficiently large, which results in a considerable relative improvement of 5.82% on word error rates.

## 1. Introduction

DNNs have been applied to several fields in recent years, among which speech recognition has been remarkably improved [1–5]. In addition, image classification [6] and natural language understanding [7] also benefit from DNNs.

A DNN model is a feedforward neural network with multiple layers which are nonlinear transformers. It transforms input vectors to corresponding output vectors and facilitates subsequent recognition. For an Automatic Speech Recognition (ASR) system, a DNN model is used to transform features of speeches to labels corresponding to states in Hidden Markov Models (HMMs) [2].

The structure of a DNN model generally consists of the following parts [2]:

- an input layer which restores the inputs from the features;
- hidden units which restore the intermediate transforming results;
- an output layer which restores the outputs representing the final transforming results;
- activation functions which simulate properties of biological neurons;
- weights which represent the importance of each unit in the input layer or the hidden layers;
- bias which provides basic points for the activation functions.

There are several methods to train DNN models, among which Gradient Descent (GD) is the most commonly used one [8]. GD uses backpropagation rules to update parameters. SGD is an improved method of GD which uses training data with randomly rearranged sequences as well as the rules of GD to train a DNN model. Based on SGD, asynchronous SGD (ASGD) raises the computing speed by parallel running tasks. ASGD on distributed systems [9] as well as on a single computer [10] has been implemented in former work. Graphic Processing Units (GPUs) are also used to improve the speed of training DNN models. Since GPUs are capable of computing matrix multiplications—which consume the largest proportion of time when training DNN models—significantly faster than CPUs, by using parallel computing technologies; they have been widely used to speed up DNN models [2]. Additionally, the stochastic data sweeping technology is also a feasible method to reduce the time consumption of training DNNs [11], which does not use all of the training data but stochastically throws some data away, based on certain rules. In addition, Hessian-free optimisation is another method [12–15] based on principles, which is different from those of GD but similar to Newton's method, to converge parameters of DNN models more quickly.

Meanwhile, some methods have been used to improve DNN models. Using restricted linear units is one of these methods [5]. Stacked denoising autoencoders have also been used to cope with noise problems on image recognition tasks [16]. To reduce the model sizes, both Singular Value Decomposition (SVD) restructuring [17, 18] and node-pruning reshaping [19] have performed well. Moreover, there is by far a new kind of DNN models that has potentials of better recognition performance and smaller model sizes, called deep Long Short-Term Memory (LSTM) neural networks [20]. These LSTM neural networks use complicated activation functions with memory to bring better performance.

In this paper, we propose another way to improve DNN models. New kinds of activation functions, namely, MSAFs, are used to replace the conventional logistic function. Experimental results reveal that DNNs with MSAFs perform better than the conventional DNNs, getting a relative improvement of 5.60% on phoneme error rates. Further experiments also reveal that mean-normalised SGD facilitates the training processes of DNNs with MSAFs, especially when being with large training sets. Furthermore, models can also be directly trained without pretraining when the training set is sufficiently large, which can get a considerable relative improvement of 5.82% on word error rates.

The rest of this paper is organised as follows. In Section 2, we first illustrate the basic forms of MSAFs and then combine them with mean-normalisation. A training method is also illustrated in this section. In Section 3, we discuss how MSAFs perform on classification tasks. In Section 4, we improve DNN models in speech recognition systems by using the MSAFs and observe their performance with different corpora. In Section 5, we draw conclusions.

## 2. Improving DNNs via the MSAFs

In this section, the basic properties of MSAFs are introduced, and these activation functions are applied into DNNs to replace the conventional activation functions and combined with mean-normalisation as well as SVD.

### 2.1. MSAFs

In DNNs of ASR systems, the conventional logistic function is one of the most widely used activation functions, called “sigmoid function” due to its shape [2]. In other words, the logistic function forms a property similar to a biological neuron, performing a resting state or an activated state. There is also a threshold point with this logistic function, similar to the threshold potential of the biological neuron. This property results in the fact that most of the units in DNNs have just two states when working. Since the two states are capable of representing two different patterns, this function is widely used to resolve classification problems.

Mathematically, the logistic function is monotonically increasing and nonlinear with the definition domain (−*∞*, +*∞*) and the range (0,1). Its expression is

(1) $$\begin{array}{c} y=\frac{1}{\left(1+e^{-x}\right)}. \end{array}$$

**Figure 1:**
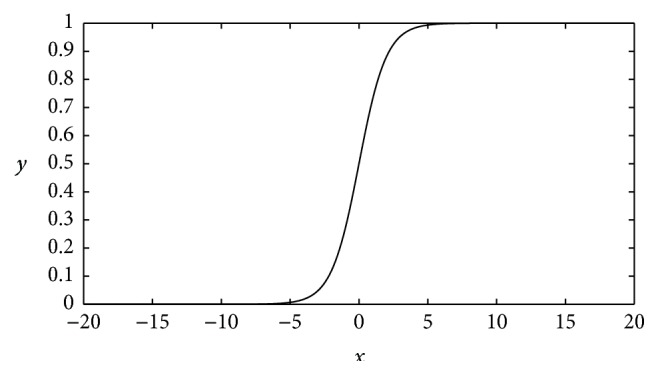
The curve of the logistic function. Its definition domain is (−*∞*, +*∞*) and its range is (0,1). This is a nonlinear function which has an exponential increase in the middle, separating state 0 and 1 apart from each other.

Figure 1 is the curve of the logistic function. It is easy to note that most of changes occur in the middle part. As *x* increases, *y* increases. The increase is significant when *x* is close to 0. By contrast, *y* is almost stable at 0 when *x* is far less than 0 and stable at 1 when *x* is far more than 0. To classify patterns, *x* can be set to the features of the patterns and the classification results are 0 or 1. The curve can also be translated by adding constants to (1), namely,

(2) $$\begin{array}{c} y=\frac{1}{\left(1+e^{-x+x_{0}}\right)}+y_{0}, \end{array}$$

where *x*_0_ and *y*_0_ are constants.

However, a DNN unit with a single logistic function can only represent two results. Increasing the number of results entails more units. In other words, to separate more complex patterns, a number of units are required, so that the model size will become larger. Revisiting Figure 1, it is noticeable that *y* represents almost the same value when *x* is far more than 0 or far less than 0, meaning that, on certain conditions, although *x*, corresponding to the features of the patterns, changes evidently, *y*, representing the classification results, almost remains unchanged, so the classification may be inefficient. To resolve this problem, extra logistic functions are added to the function to form new MSAFs. For instance, the 2-order MSAF is

(3) $$\begin{array}{c} y=\frac{1}{\left(1+e^{-x}\right)}+\frac{1}{\left(1+e^{-x+C}\right)}, \end{array}$$

where *C* is a constant.

**Figure 2:**
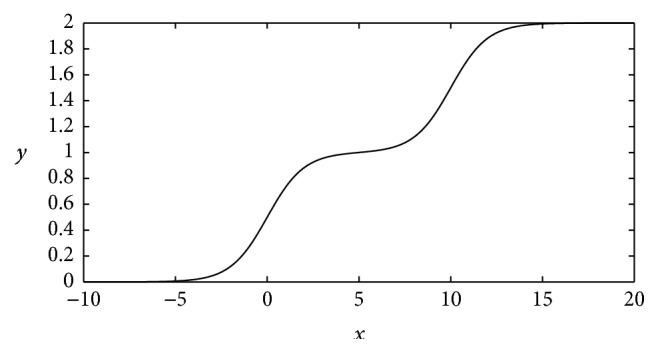
The curve of the 2-order MSAF. It has 3 states, including the conventional 0 and 1 as well as an extra 2.

Figure 2 shows the curve of the 2-order MSAF. In comparison with Figure 1, it has an extra logistic function at its right part, whose effect is that when *x* is close to *C*, a reclassification occurs. Thus, this function will reach state 2 if *x* is sufficiently big. Meanwhile, this function can also reach state 1, because there is a stable part in the middle. When *x* is small, it also has state 0. To sum up, this function separates a pattern into three classes.

Furthermore, MSAFs will have more states if more logistic functions are added. The general presentation of MSAFs is

(4) $$\begin{array}{c} y=y_{0}+\overset{N}{\underset{i=1}{\sum }}\frac{1}{\left(1+e^{-x+x_{i}}\right)}, \end{array}$$

where *y*_0_ ∈ *ℝ*, *N* ∈ *ℕ* ^+^, *x*_*i*_ ∈ *ℝ* ^+^, and *x*_1_ < *x*_2_ < ⋯<*x*_*N*_. When *y*_0_ = 0, we name it an *N*-order MSAF. In DNN models, if the backpropagation algorithm is used, the derivative function of MSAFs is needed, which is

(5) $$\begin{array}{c} \frac{dy}{dx}=\overset{N}{\underset{i=1}{\sum }}\frac{e^{-x+x_{i}}}{{\left(1+e^{-x+x_{i}}\right)}^{2}}. \end{array}$$

In addition, there is a special MSAF worthy to be singled out, called the symmetrical MSAF, which is

(6) $$\begin{array}{c} y=-1+\frac{1}{\left(1+e^{-x-x_{1}}\right)}+\frac{1}{\left(1+e^{-x}\right)}, \end{array}$$

where *x*_1_ is required to be markedly smaller than 0.

**Figure 3:**
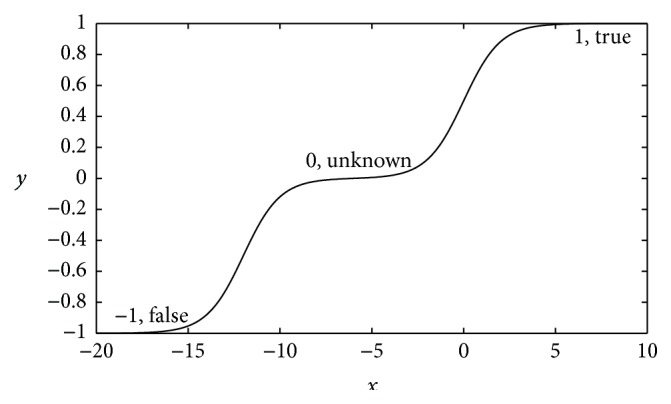
The curve of the symmetrical MSAF. It has a couple of symmetrical states −1 and 1, as well as state 0. It can also be used to represent trivalent logic.

Figure 3 reveals the curve of the symmetrical MSAF. It is symmetrical as it has the symmetrical states −1 and 1. Combining with state 0, it forms a special activation function including three states. If neural networks are used to deal with logic problems, this activation function will be helpful on some certain conditions. For instance, on trivalent logic, it can directly represent “false” as −1, “unknown” as 0, and “true” as 1, whereas, on classification problems, this activation function allows some units to temporarily have almost no contribution to whole DNN models. In other words, these units “hide” themselves by keeping their states at 0, and the other units, which have states at −1 or 1, are predominant in the whole models.

### 2.2. Mean-Normalisation for MSAFs

The basic concept of mean-normalisation is to make the running average of each unit be close to 0 as near as possible. During the forward pass of the backpropagation algorithm, the algorithm counts the running average of each unit, and during the backward pass it updates the parameters of DNN models via mean-normalised SGD, as proposed in [21].

Let Δ*W* and Δ*b* denote the deltas of the weight matrix and bias computed by conventional SGD, respectively, and *a* denotes the running averages. During the backward pass, the delta of the weight matrix is (“MN” denotes mean-normalisation)

(7) $$\begin{array}{c} \Delta W_{MN}=\Delta W-a\cdot \Delta b^{T}, \end{array}$$

and the delta of the bias is

(8) $$\begin{array}{c} \Delta b_{MN}=\left(\;1+a^{T}\cdot a\;\right)\cdot \Delta b-\Delta W^{T}\cdot a. \end{array}$$

The running average *a* is

(9) $$\begin{array}{c} a=\left(\;1-\alpha \;\right)\cdot a_{pre}+\alpha \cdot a_{this}, \end{array}$$

where *a*_pre_ denotes the previous running average and *a*_this_ denotes the current average value of the units in DNNs and *α* denotes the smoothing factor which is a very small real number.

Although not necessary, it is significant to combine MSAFs with the mean-normalised rules. Since MSAFs have more than 2 different states, it is important to restrict their running averages in a certain range to ensure that the multistates are used as much as possible. Using the mean-normalised rules, the running averages can be restricted to somewhere near 0.

**Figure 4:**
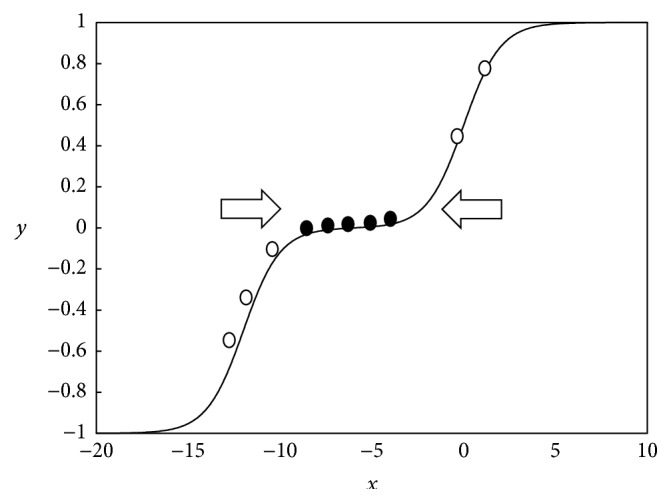
Restricting running averages to somewhere near 0. The hollow points stand for running averages of the conventional SGD, while the solid points stand for running averages of mean-normalised SGD.

Figure 4 shows the effectiveness of mean-normalisation on the symmetrical MSAF. If the running averages are not mean-normalised, they will distribute in a relatively large district. On the other hand, if mean-normalised SGD is applied, the running averages will be restricted to approximately 0. It is worthy to note that 0 means “unknown” states for the symmetrical MSAF. As mentioned before, this state means that a unit temporarily hides itself. Therefore, mean-normalisation allows more units to have the “unknown” states and consequently prevents the model from being impacted by disturbances. As for the other MSAFs which are not symmetrical, mean-normalisation is also useful, as it provides relatively stable basic points. To be concrete, a relatively stable point means where a small disturbance does not bring about an evident influence, whereas a relatively unstable point means where a small disturbance will cause an evident change to the output. The points near 0 are relatively stable as the gradients of these points are relatively small.

### 2.3. Learning Models without Pretraining

Pretraining is very important for learning DNNs [22]. When the number of hidden layers increases, DNNs are faced with the vanishing gradient problem [23], which is serious when the training set is small. Pretraining can be used to resolve this problem. However, in some case, pretraining is not necessary [24].

When DNNs are pretrained using MSAFs, they are not optimal due to the fact that the activation function of a restricted Boltzmann machine (RBM) is different from MSAFs. The difference of the activation functions between pretraining and training may make the final performance of the model worse. Although we could further seek a method of replacing the activation function of RBMs by one of MSAFs, it is possible that MSAFs are detrimental to the entire model, because optimization on RBMs is based on the logistic function or the rectified linear unit, which may not be used to other kinds of activation. One way to resolve the problem is to directly train a model without pretraining. In this case, the model prevents itself from becoming worse because it uses the same activation function from the beginning to the end of training.

DNNs with MSAFs can be trained without pretraining provided that the training set is sufficiently big. However, in our early work, we found that mean-normalised SGD is faced with a more serious gradient vanishing problem than the conventional SGD. In other words, it is harder for mean-normalised SGD to make the parameters converge than the conventional SGD. Besides, this problem becomes more considerable when mean-normalised SGD is used to train DNNs with MSAFs. Nevertheless, there is a feasible way to resolve this problem. Since mean-normalised SGD uses the 2-order rules based on the 1-order rules, it is workable to use the latter to update the model before using the former, which will not bring negative impacts to the model. Concretely, the following method can be used to train the models, provided that the mean-normalised SGD cannot directly make the parameters converge:

(i) Step 1: training a DNN model via the conventional SGD until reaching a suitable accuracy;
(ii) Step 2: training the obtained model via mean-normalised SGD.

Reaching a “suitable accuracy” means where the parameters can certainly converge. Practically, it is easy to measure whether or not the parameters can converge, because there will be a boosted accuracy during the first or second epoch of the training iterations if the parameters are able to converge, whereas the accuracy cannot be seen if the parameters cannot converge. In other words, the cross entropy loss, which is usually used to measure the accuracy, can reveal the moment after one or two epochs.

Another important facet of training a model is the learning rate. A glance at the curves of the MSAFs reveals that they have at least two slopes, whereas the logistic function just has one, meaning that MSAFs make the gradients become larger, leading to more significant changes for parameters when training models. Therefore, the learning rate for MSAFs should be smaller than that for the logistic function, and the more states the MSAFs have, the smaller the learning rate should be.

## 3. A Closer Look at Classification Problems

In this section, we closely observe how MSAFs perform when they are used to classify patterns. First, we observe the *N*-order functions and then the symmetrical function.

**Figure 5:**
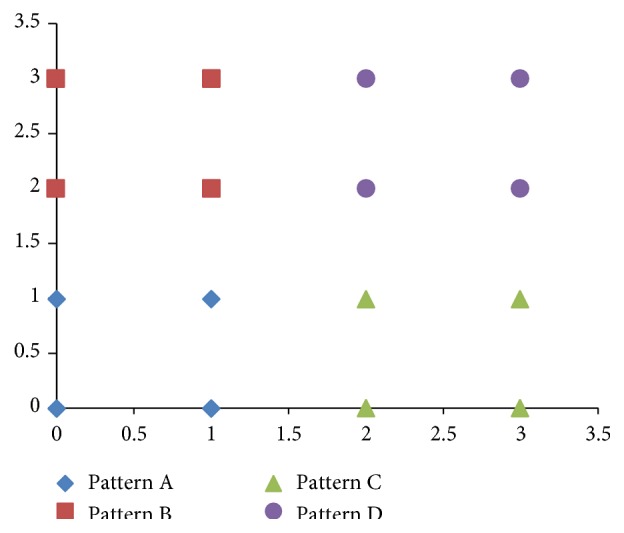
An example of classification. The primary task is to classify these 4 patterns and mark them, respectively.

Figure 5 illustrates a classification task. There are four different patterns in this figure. Each pattern corresponds to two values, which are its horizontal and longitudinal coordinates, respectively. According to their coordinates, they will be divided into four different classes. If we use a neural network and the logistic function to classify them, the neural network will have a considerable number of units, especially in its output layer, due to the fact that the logistic function only has two states.

**Figure 6:**
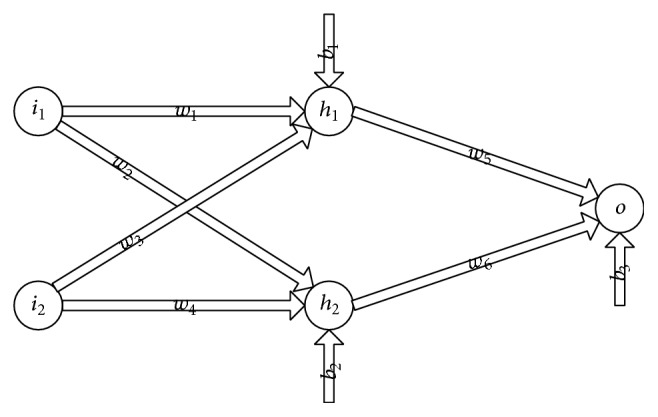
A neural network. In this model, *i*_1_ and *i*_2_ are input units; *h*_1_ and *h*_2_ are hidden units; *o* is an output unit; and *w*_*i*_ and *b*_*i*_ are weights and bias, respectively.

However, if MSAFs are used, the number of units can be reduced because MSAFs have more than two different states.  Figure 6 provides a neural network which will be used to resolve the problem. In this neural network, the hidden units and the output unit use MSAFs as their activation functions. The input units of this neural network correspond to the horizontal and longitudinal coordinates, respectively, and the output unit will provide classifying results. There are a variety of different combinations of weights, bias, and activation functions which are able to classify the patterns. For instance, let (*w*_1_, *w*_2_, *w*_3_, *w*_4_, *w*_5_, *w*_6_, *b*_1_, *b*_2_, *b*_3_) = (−16, −32,48,16,40, −48, −40,40,32) and let the activation functions of the hidden units and the output unit be a 3-order MSAF: *y* = 1/(1 + *e* ^−*x*^) + 1/(1 + *e* ^−*x*+20^) + 1/(1 + *e* ^−*x*+40^). Then, the output unit will sign the patterns as

- Pattern A—0;
- Pattern B—1;
- Pattern C—2;
- Pattern D—3.

The patterns thus are divided into 4 different classes. In fact, not only these values of weights and bias but also some other values are able to achieve this goal. For instance, (−16,40,40, −8,16,24, −24, −24, −40) and (−8,40,48,0, 8,16, −40, −40, −16) are also workable.

**Figure 7:**
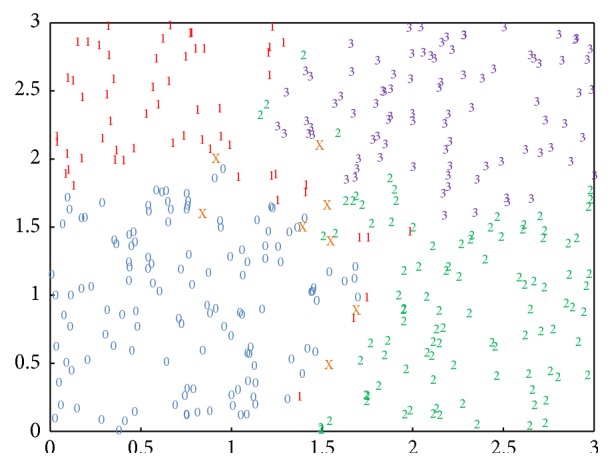
Results of classifying random points on the coordinate plane. The inputs to the neural network are the coordinates of the random points. The numbers on the coordinate plane reveal the output states. Different output states are marked by different colours.

The neural network is capable of classifying not only certain points in Figure 5 but also the other points near these certain points.  Figure 7 provides the classification results of 400 random points. A glance at this figure reveals that the points are classified into 4 different certain groups, denoted by “0,” “1,” “2,” and “3,” respectively, as well as an uncertain group, denoted by “X.” (If an output is very close to an integer, the integer can be directly used to mark it. Let *o* denote the output state and let abs(*x*) denote the absolute value of *x*. In Figure 7, a point is marked as “0” if abs(*o* − 0) < 0.1, “1” if abs(*o* − 1) < 0.1, “2” if abs(*o* − 2) < 0.1, and “3” if abs(*o* − 3) < 0.1. The other cases are marked as “X,” meaning that these outputs are not very close to any integer.) It is noticeable that the coordinate plane is divided into 4 districts, dominated by certain numbers and coloured by blue, red, green, and purple, respectively. Under these circumstances, only a few points are mistakenly marked. Moreover, most of the uncertain points are in the margins of the districts.

These results reveal that MSAFs are capable of classifying patterns into more than 2 groups using only one output unit. In fact, the above instance is just a simple task and there is another small but more important problem.

*A 2-Order Table of Values for Mapping the Neural Network.* It is based on the binary XOR problem

(10) 012012012101210

Equation (10) is a table based on the binary XOR problem [25]. The numbers outside the boxes are inputs and the ones inside the boxes are expected outputs. It is noticeable that the rows and columns including only 0 and 1 actually form the truth table of the binary XOR. The primary task is to find a set of parameters for the neural network that can transform the inputs to the corresponding outputs.

*A 3-Order Table*. Consider

(11) 012301230123101221013210

It is easy to use the neural network illustrated by Figure 6 and the logistic function to resolve the binary XOR problem [25]. However, it is impossible for them to represent (10) due to the fact that the logistic function does not have state “2.” Nevertheless, it is possible to use MSAFs to resolve it. Using the 3-order MSAF, the neural network is able to represent the values with sets of parameters of (*w*_1_, *w*_2_, *w*_3_, *w*_4_, *w*_5_, *w*_6_, *b*_1_, *b*_2_, *b*_3_), such as (−24,16,24, −16,16,16, −8, −8, −8) and (16, −16, −16,16,16,24,16, −8, −24).

Further, if the table is extended to 4 rows with 4 columns as shown in (11), we can also find parameters to represent the values without changing the structure of the neural network. For instance, let (*w*_1_, *w*_2_, *w*_3_, *w*_4_, *w*_5_, *w*_6_, *b*_1_, *b*_2_, *b*_3_) = (24, −24, −24,24,24,24, −16, −16, −16); the network will work.

**Figure 8:**
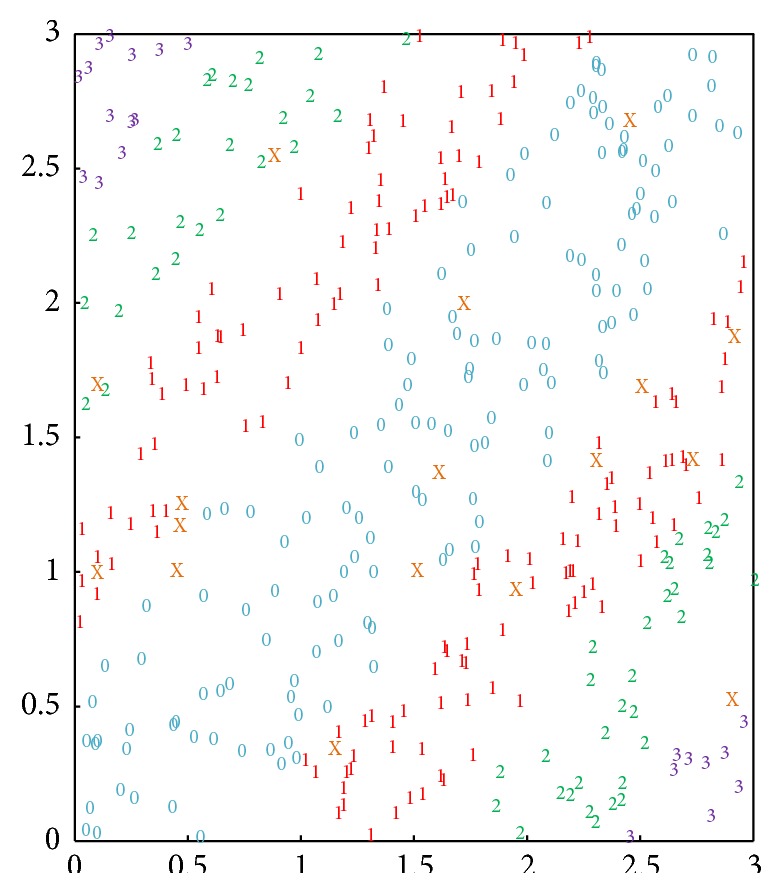
Results of classifying random points by using the neural network based on the binary XOR. The points are separated into 4 classes and 7 districts.

The neural network can also classify the other points, as illustrated in Figure 8. The coordinate plane is separated into 4 classes and 7 districts approximately. A glance at the figure reveals that these districts are symmetrical for the diagonal of the plane. Although the numbers of the points are different in terms of the classes, the widths are almost the same. More importantly, the results are quite different from those in Figure 7, as the points marked by the same number can be in two districts which are far away from each other.

**Figure 9:**
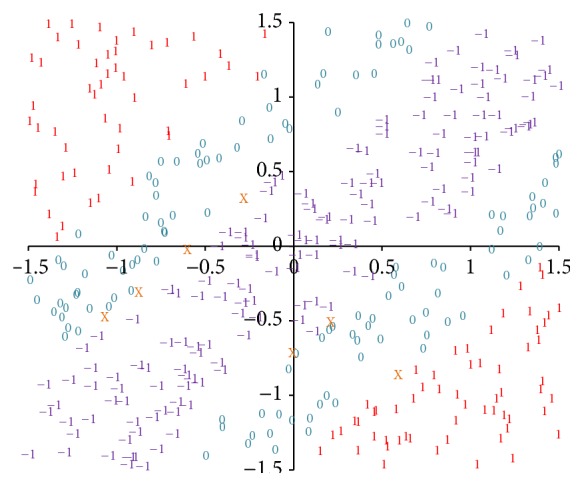
Results of classifying random points using the symmetrical MSAF. The results contain positive states and zero states as well as negative states.

Using the symmetrical MSAF, the neural network is able to classify negative values and output negative states as well. For instance, let the activation function be *y* = −1 + 1/(1 + *e* ^−*x*−20^) + 1/(1 + *e* ^−*x*^) and (*w*_1_, *w*_2_, *w*_3_, *w*_4_, *w*_5_, *w*_6_, *b*_1_, *b*_2_, *b*_3_) = (−24, −24,24,24, −40,32,16, −32,24); then the network will classify random points shown in Figure 9. It is easy to note that the inputs and the hidden units both contain negative coordinates. In fact, the symmetrical MSAF enables the neural network to have positive and negative states at the same time.

On the other hand, it is noticeable that the neural network in Figure 6 only has a single output unit. The concept of this neural network is different from the binary neural network which uses multioutput units to solve multiple classification problems. Concretely, to classify numeric attributes into *N* different classes by using a binary neural network, at least *N* − 1 output units are required. A numeric attribute is classified into the *i*th class when the (*i* − 1)th unit has a positive state and is classified into 0 class when none of these units have a positive state [26] (see pages 338-339 of this reference). The neural network with MSAFs is different from this method, which represents different classes using only a single output unit. Admittedly, a single output unit with an *N*-order MSAF is weaker than *N* binary output units but is stronger than a single binary output unit.

In summary, MSAFs enable the neural network to separate the patterns into more than two classes, whereas the logistic function can only separate two classes, so MSAFs are more powerful when resolving classification problems.

## 4. Experiments

The experiments are conducted on a server with 4 Intel Xeon E5-2620 CPUs and 512 GB memories. An NVIDIA Tesla M2070-Q graphics card is used to train DNN models. The experiments consist of two parts. Part 1 tests the performance of the basic MSAFs on the standard TIMIT corpus. Part 2 tests the combination of MSAFs with mean-normalisation on a relatively big corpus.

### 4.1. Experiments on TIMIT

First, we use Mel Frequency Cepstrum Coefficient (MFCC) as basic features. GMM models with 2000 senones are trained by applying Monophone, Triphone, Linear Discriminant Analysis (LDA), and Maximum Likelihood Linear Transformation (MLLT) in turn, based on the Kaldi speech recognition toolkit [27]. The alignments of the GMM models are then used to train DNN models. Then, deep belief nets including 6 hidden layers and 1,024 units in each hidden layer are pretrained by the unsupervised training algorithm proposed in [22]. Next, on the basis of the deep belief nets, DNN models with different activation functions are trained via SGD. (We use the conventional learning rate strategy. That is, an initial learning rate is used at first. When the improvement of the cross entropy loss (on the cross-validation set) is smaller than a certain value, the learning rate starts halving. When the improvement of the cross entropy loss is smaller than a very small value, the training process stops.) Finally, phoneme error rates (PERs) of these models are tested, using both the development set and the test set.

Table 1 provides the experimental results on TIMIT. The lowest cross entropy loss on the training set occurs when the 2-order MSAF is used, whereas the lowest one on the cross-validation set occurs when the symmetrical MSAF is used. It is also worthy to note that, on the cross-validation set, the cross entropy loss of the symmetrical MSAF is just slightly higher than that of the 2-order MSAF. The cross entropy losses of the 3-order MSAF are higher than those of the other two MSAFs but lower than those of the logistic function. Table 1 reveals that MSAFs perform better than the logistic function in terms of a single DNN model. With regard to PERs, the 2-order MSAF sees the best result of 21.8% on the development set, and the relative improvement comparing with the conventional logistic function achieves 5.22%. On the test set, the symmetrical MSAF sees the best result of 23.6%, and the relative improvement is 5.60%. It is also noticeable that all MSAFs in the experiments have better PERs than the conventional logistic function, which means MSAFs have better performance than the logistic function in the whole speech recognition system.

### 4.2. Further Experiments

The following experiments use a speech database consisting of approximately 30-hour training data and 3-hour testing data with their transcriptions. The language model is a 5-gram ARPA model.

First, GMM models including 5,000 senones are trained by applying Monophone, Triphone, LDA, and MLLT in turn, using MFCC as features. The alignments of the GMM models and the Feature-Space Maximum Likelihood Linear Regression (FMLLR) are then used to train the DNN models. The DNN models are pretrained before supervised training and subsequently trained by conventional SGD or mean-normalised SGD. A single DNN model in the experiments has 6 hidden layers, and each hidden layer has 1,024 units. The input layer has 440 units and the output layer has 3,998 units with softmax as their activation function. After that, the SVD restructuring method illustrated in previous works [17, 18] is used to reduce the model sizes. The number of kept singular values is set to 256. Finally, word error rates (WER) of these models are tested (the test dataset contains 17,221 words totally).

Table 2 provides the experimental results of the cross entropy losses on the training set. The logistic function sees the lowest loss in terms of plain training, whereas the 2-order MSAF has the highest, revealing that the conventional training method does not perform well when the MSAFs are applied. However, the training method with mean-normalisation sees contrary phenomena. The loss of the logistic function task is slightly higher if the model is trained by mean-normalised SGD, while the losses of the MSAF tasks drop considerably. Furthermore, all of the losses of the MSAF tasks decrease to somewhere lower than that of the logistic function task, which means that the models with MSAFs now have higher fitting degrees with the training data. After being restructured by the SVD method, the losses of these models increase evidently, but the model sizes have shrunk. Under these circumstances, the loss of the logistic function task is still the highest one.

Table 3 provides the cross entropy losses on the cross-validation set. Obviously, the symmetrical MSAF has the best cross entropy losses. In addition, the losses stay low after SVD restructuring, which means that most information loss caused by SVD is corresponding to the training data, and the fitting degrees between the models and the cross-validation set are not evidently impacted by SVD.

Table 4 provides WERs of all tasks. We can see that the logistic function gets the best WER when the plain training method is applied. Nevertheless, the WERs of the MSAF tasks reduce significantly if mean-normalisation is applied, whereas the WER of the logistic function task increases. After the SVD method is used, the WERs of the 2-order MSAF task and the 3-order MSAF task rise slightly, but they are still lower than those of the logistic function task, and meanwhile the model sizes have been largely reduced. It is also worthy to note that the WER of the symmetrical MSAF task with mean-normalisation is also lower than that of the logistic function tasks.

A glance at Tables 2 and 4 reveals a similarity between them. The two tables show that the model performance of the logistic function task becomes worse if trained by mean-normalised SGD, while the model performances of the MSAFs become evidently better. Thus, we get a conclusion:* mean-normalisation facilitates the training of DNN models with MSAFs*.

In comparison with the experiments on TIMIT, the amount of the training data in this subsection is evidently larger. Since a relatively small training set will have less impact on the running average, MSAFs still perform well though mean-normalisation is not used.

### 4.3. Experiments Using a Big Training Set

The DNN models can be trained without pretraining if the training set is sufficiently big. In this subsection, we use a 120-hour training set to train the models. In our earlier work, we have found that this training set is able to train a model with 5 hidden layers but not able to train a model with 6 hidden layers. Therefore, models with 5 hidden layers and 1,024 units in each hidden layer are used during the following experiments. The initial stochastic models with different activation functions will be directly trained via the plain SGD or the mean-normalised SGD. To ensure that the models trained by the mean-normalised SGD are able to converge, the method in Section 2.3 is applied to the training processes. Concretely, the models will be trained via the mean-normalised SGD after one epoch of the plain SGD. Finally, the WERs of these models are tested.

**Figure 10:**
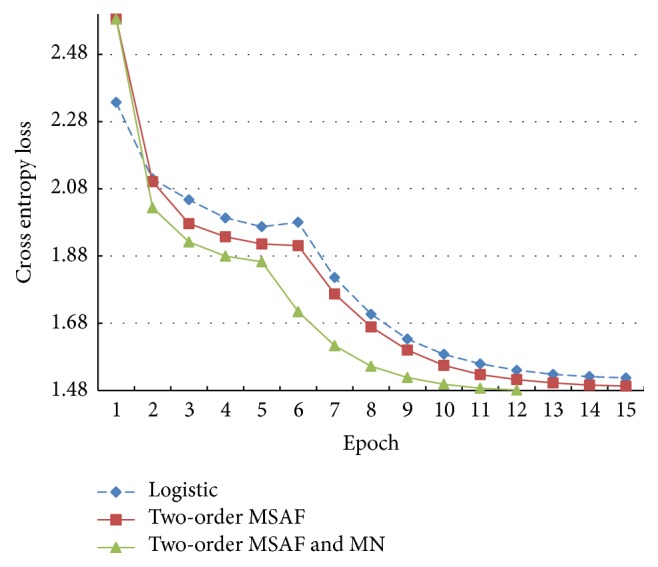
The cross entropy losses of the models using the 2-order MSAF on the cross-validation set. The curve of the logistic function is set to make the comparison clearer.

Figure 10 provides curves of the cross entropy losses when the models use the 2-order MSAF as their activation functions as well as a curve of the logistic function. It is obvious that the 2-order MSAF has better results than the logistic function, and the results are considerably better when mean-normalisation is applied. Although both the logistic function and the 2-order MSAF require 15 epochs of iterations to finish training, the final cross entropy loss of the latter is slightly lower than that of the former. Also noteworthy is that the 2-order MSAF combined with mean-normalisation sees the lowest loss and it requires only 12 epochs of iterations.

**Figure 11:**
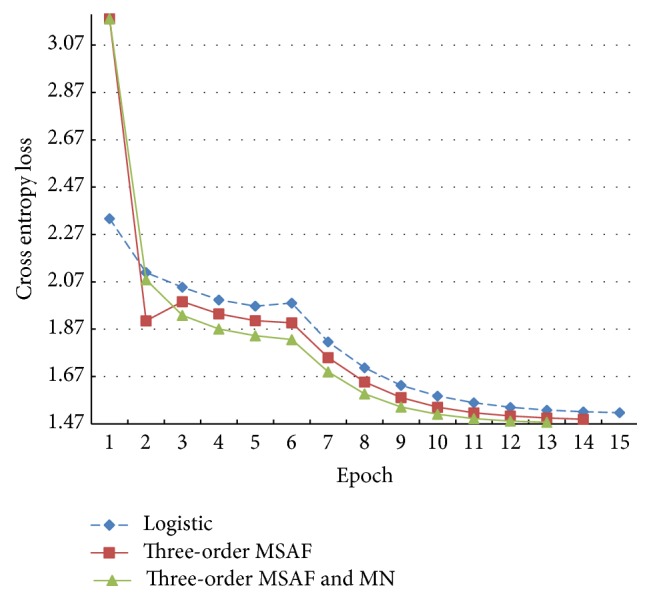
The cross entropy losses of the models using the 3-order MSAF on the cross-validation set.

Figure 11 provides curves of the cross entropy losses when the models use the 3-order MSAF as their activation functions as well as a curve of the logistic function. The loss of the MSAF during the first epoch is evidently higher than that of the logistic function. However, it becomes better after the second epoch. The MSAF obtains lower final losses than the logistic function, and the mean-normalisation further brings about the lowest one. Moreover, the MSAF also requires less epochs of iterations than the logistic function in this part of experiments.

**Figure 12:**
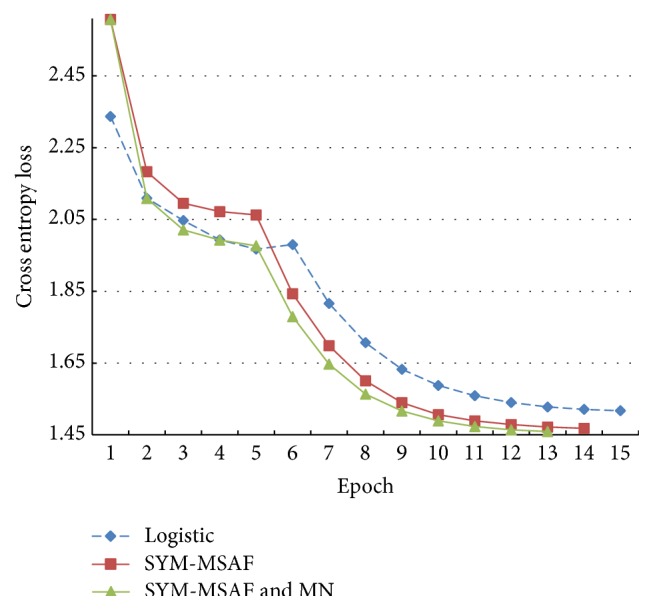
The cross entropy losses of the models using the symmetrical MSAF on the cross-validation set.

Figure 12 provides curves of the cross entropy losses when the models use the symmetrical MSAF as their activation functions as well as a curve of the logistic function. At the beginning, the symmetrical MSAF does not have better performance than the logistic function. Nevertheless, it performs better when the cross entropy losses drop significantly during the sixth epoch. The symmetrical MSAFs both without and with mean-normalisation see considerably lower cross entropy losses at the end of training, and the one with mean-normalisation performs slightly better than the other.

With further studies on Figures 10, 11, and 12, we can find that the symmetrical MSAF with the mean-normalisation has best results. Another important fact is that the mean-normalisation decreases the time consumption and improves the models.

Table 5 provides final WERs and the *t*-test results of the models. It is noticeable that MSAFs bring about better WERs than the logistic function, and the mean-normalisation further improves the models. The symmetrical MSAF with the mean-normalisation sees the best WER in all of these experiments, and the relative improvement is approximately 5.82% when it is compared with the conventional logistic function. Without the mean-normalisation, the symmetrical MSAF gets the relative improvement of around 3.05%. Both the 2-order MSAF and the 3-order MSAF with the mean-normalisation get the relative improvement of 2.77% as well. The *t*-test significance has also been tested, as the improvements of WERs are not significant. The samples for *t*-test are the relative improvements of the WERs on different language model weights (the weights are 6,7, 8,…, 15; therefore, there are 10 samples for each result), in comparison with the conventional logistic function. The significance of the 2-order MSAF is evidently greater than 0.05, revealing that the possibility that the 2-order MSAF does* not* really bring improvements for the model is high. However, the significance of the other functions is smaller than 0.05, revealing that it is highly possible that these functions really bring improvements for the model.

## 5. Conclusions

In this paper, MSAFs are proposed and used to improve the DNN models. The basic MSAFs can be formed with addition of several logistic functions and a constant. There are two kinds of MSAFs outlined, the *N*-order MSAF and the symmetrical MSAF. Combined with MSAFs, the DNN models can then be trained by mean-normalised SGD. Furthermore, MSAFs have more powerful abilities of classifying patterns because a single MSAF is able to output more states than the logistic function.

On speech recognition tasks, MSAFs improve the DNN models when the training set is small and improve the DNN models with mean-normalised SGD when the training set is large. In fact, mean-normalisation evidently facilitates the training processes of the DNN models with MSAFs. More importantly, the models with MSAFs can also be trained without pretraining but still perform better than the conventional models.

## Figures and Tables

**Table 1:** Experimental results on TIMIT. “TR-XENT” means the cross entropy loss on the training set. “CV-XENT” means the cross entropy loss on the cross-validation set. “DEV-PER” means the PER on the development set. “TEST-PER” means the PER on the test set. “SYM-MSAF” means the symmetrical MSAF.

Activation function	TR-XENT	CV-XENT	DEV-PER (%)	TEST-PER (%)
Logistic	2.07	2.45	23.0	25.0
Two-order MSAF	**1.89**	2.29	**21.8**	24.0
Three-order MSAF	1.97	2.30	22.5	24.3
SYM-MSAF	1.91	**2.24**	22.1	**23.6**

**Table 2:** Cross entropy losses on the training set. “Plain” means the conventional SGD without mean-normalisation and SVD restructuring. “MN” means mean-normalised SGD and “MN + SVD” means the SVD restructuring method using mean-normalised SGD.

Activation function	Plain (%)	MN (%)	MN + SVD (%)
Logistic	1.4466	1.4602	1.5401
Two-order MSAF	1.5401	**1.4183**	1.5005
Three-order MSAF	1.4682	**1.4176**	1.5002
SYM-MSAF	1.4637	**1.4373**	1.5177

**Table 3:** Cross entropy losses on the cross-validation set.

Activation function	Plain (%)	MN (%)	MN + SVD (%)
Logistic	1.7793	1.7648	1.7688
Two-order MSAF	1.7726	1.7743	1.7716
Three-order MSAF	1.7926	1.7733	1.7720
SYM-MSAF	**1.7597**	**1.7539**	**1.7608**

**Table 4:** WERs of the models.

Activation function	Plain (%)	MN (%)	MN + SVD (%)
Logistic	**17.26**	17.43	17.73
Two-order MSAF	17.43	**16.97**	**17.11**
Three-order MSAF	17.55	**16.95**	**17.10**
SYM-MSAF	17.51	**17.07**	17.50

**Table 5:** Final WERs and the *t*-test results of the DNNs without pretraining.

Activation function	WER (%)	*t*-test significance
Logistic	14.44	/

Two-order MSAF	14.31	2.516 × 10^−1^
Three-order MSAF	14.14	3.460 × 10^−7^
SYM-MSAF	**14.00**	1.304 × 10^−5^

Two-order MSAF and MN	**14.04**	5.925 × 10^−6^
Three-order MSAF and MN	**14.04**	8.166 × 10^−7^
SYM-MSAF and MN	**13.60**	7.111 × 10^−9^
